# Theoretical design of multi-colored semi-transparent organic solar cells with both efficient color filtering and light harvesting

**DOI:** 10.1038/srep07036

**Published:** 2014-11-13

**Authors:** Long Wen, Qin Chen, Fuhe Sun, Shichao Song, Lin Jin, Yan Yu

**Affiliations:** 1 Key Lab of Nanodevices and Applications-CAS & Collaborative Innovation Center of Suzhou Nano Science and Technology, Suzhou Institute of Nano-Tech and Nano-Bionics, Chinese Academy of Sciences (CAS), Suzhou 215123, P. R. China; 2 State Key Laboratory on Integrated Optoelectronics, Institute of Semiconductors, Chinese Academy of Sciences, Beijing 100083, China

## Abstract

Solar cells incorporated with multi-coloring capability not only offer an aesthetic solution to bridge the gap between solar modules and building decorations but also open up the possibility for self-powered colorful display. In this paper, we proposed a multi-colored semi-transparent organic solar cells (TOSCs) design containing metallic nanostructures with the both high color purity and efficiency based on theoretical considerations. By employing guided mode resonance effect, the multi-colored TOSC behave like an efficient color filter that selectively transmits light with the desired wavelengths and generates electricity with light of other wavelengths. Broad range of coloring and luminosity adjusting for the transmission light can be achieved by simply tuning the period and the duty cycle of the metallic nanostructures. Furthermore, accompanying with the efficient color filtering characteristics, the optical absorption of TOSCs was improved due to the marked suppression of transmission loss at the off-resonance wavelengths and the increased light trapping in TOSCs. The mechanisms of the light guiding in photoactive layer and broadband backward scattering from the metallic nanostructures were identified to make an essential contribution to the improved light-harvesting. By enabling efficient color control and high efficiency simultaneously, this approach holds great promise for future versatile photovoltaic energy utilization.

In the past decades, scientists are constantly seeking different light trapping and material upgrading strategies that aim to improve the efficiency of solar panels^1^,^2^,^3^,^4^,^5^,^6^,^7^. Recent advances in integration of photovoltaic electricity into building facades, rooftops, solar windows^8^,^9^,^10^,^11^,^12^ and self-powered electronic devices^13^,^14^ may offer a new direction for future photovoltaic energy utilization. Among them, visibly transparent organic solar cells (TOSCs) are extremely attractive because of their light weight, flexibility, cost-effective production and inherent semi-transparent properties^15^,^16^,^17^,^18^. As compared with the mainstream of inorganic materials research in solar cells, the photoactive organic materials of TOSCs have relatively flat-band absorption coefficient above their band edge. This means that when the opaque metallic electrode is removed, the TOSCs with inefficient single-pass absorption suffer from transmission loss over a broad spectral range. As a consequence, the transparency of TOSCs is always accompanied by seriously undermined conversion efficiency. While the conversion efficiency for the single-junction opaque counterparts has steadily been improved close to 10%^19^,^20^,^21^, TOSCs decrease it nearly in half to around 5% due to their compromised light collection capabilities^15^,^16^,^17^.

TOSCs with tunable transmission colors have the advantages of being more aesthetically pleasing, multi-functional and ease of integration with decorative building elements or electronic display devices^18^,^22^. However, the color perceptions obtained in TOSCs depend heavily on the absorption characteristics of the photoactive layer. The large band gap polymers based TOSCs like the poly(3-hexylthiophene) (P3HT):[6,6]-phenyl-C_61,71_ butyric acid methyl ester (PC_61,71_BM) blend combinations absorb strongly above their band edge and transmit large amounts of light at the red-end of the visible spectrum, thereby resulting in reddish or brownish colors^17^,^23^. The low band gap polymers have relative flat and broad absorption band extending to near-infrared and possess almost neutral light transmission. Slight color casts closed to the white point are reported for these systems with the typical active layer thickness around 100 nm^15^,^16^,^23^,^24^. Increasing the thickness of these low band gap polymers can improve the purity of the color perceptions but leads to low luminosity and compromised electrical performance^23^. Although the inherent color perceptions of TOSCs can be changed by incorporating different dyes, this approach would be less desirable due to the energy losses from the complementary absorption in the dyes^24^. In contrast, structural colors produced by the interaction of light with structures are more suitable for designing TOSCs with color tunability across the entire visible spectrum, through precise tailoring of the structures. Multilayered thin-film design that utilizes the interference effect was previously used to achieve spectral engineering in the TOSCs for the purpose of recycling near-infrared/ultraviolet photons^17^,^18^ or selectively transmitting light with specific colors^22^. By incorporating one-dimensional aperiodic photonic crystal stacks in the rear side of TOSCs, Betancur et al. demonstrated the possibility to tune the color of the device with different relative layer thicknesses of the stacks^18^. However, the colored cells exhibited similar transmission curves without obvious monochromatic features in the visible, thus leading to poor color purity. Lee et al. proposed a multi-colored hybrid organic-inorganic solar cells design by employing constructively interference in multilayer stacks^22^. To achieve color tunability, inorganic absorbing layers with only 6 nm, 11 nm, and 31 nm were selected for blue, green and red cells. Although, monochromatic coloring and near unity internal quantum efficiency were obtained, their efficiencies were significantly undermined by the poor light-harvesting capacity for such ultrathin absorbing layers.

The aim of this research is to find a strategy that allows the TOSCs to provide more aesthetically pleasing colors and efficient color tunability, on the premise of maintaining high conversion efficiencies comparable to their opaque counterparts. Additive color filtering schemes^25^,^26^,^27^,^28^,^29^,^30^,^31^ that selectively transmit the desired wavelengths in a well-defined narrow-band have the potential to be used in TOSCs for the purpose of achieving high color purity while preserving efficient light harvesting capacity. In this work, two-dimensional metallic nanostructures were employed as guided mode resonance (GMR) couplers to obtain desirable colored semi-transparency for the TOSCs. The key advantage of the proposed approach is that the TOSCs allow only the light on resonance to pass through and contribute to the colored semi-transparency, but absorbing all the rest for electricity generation via light guiding and plasmonic backscattering effects. The rest of the paper is organized as follows. Firstly, we outline the additive filtering characteristics based on the one-dimensional (1D) metallic grating architectures. The underlying physics is clarified by analyzing the dispersion relationships and modal profiles for the photonic mode supported by the multi-layered stacks. Secondly, we present the multi-colored TOSCs containing 2D metallic nanodiscs and evaluate their color tunability by calculating the CIE 1931 xy chromaticity coordinates for different geometries. Thirdly, we discuss the absorption performance of the multi-colored TOSCs and identify the origins of the increased light trapping capacity. Fourthly, we investigate the relationship between the color control, luminosity and the light harvesting of the multi-colored TOSCs to illustrate how to design such a high-performance multi-colored TOSC. Using the similar design frameworks, we demonstrate that the inverted design can still remain in force, guaranteeing the efficient color control, clear color perceptions and light harvesting. Finally, the conclusion is given.

In order to obtain the color transparency throughout the entire visible region for the TOSCs, it is essential to choose an organic photoactive layer that has relative flat-band absorbability and a small bandgap with invisible sub-bandgap light transmission. In other words, material without any obvious inherent color perception is preferred in designing of TOSCs with high saturation and tunable structural colors. Thus, in this work, the conjugated organic polymer blends of *poly[2,6-(4,4-bis-(2-ethylhexyl)-4H-cyclopenta[2,1-b;3,4-b0]dithiophene)-alt-4,7-(2,1,3-benzothiadia-zole):phenyl-C_71_-butyric acid methyl ester* (PCPDTBT- PC_70_BM) was used as the absorber layer because of its photoactive region is extended into the near infrared (300 nm–900 nm)^23^,^32^,^33^. The schematic of our one-dimensional (1D) numerical model for the multi-colored TOSCs is shown in Figure 1. The device configuration is glass substrate/ITO anodes/PEDOT:PSS HTL/PCPDTBT-PC_70_BM active layer/DMD cathodes/Ag gratings. The thickness of the PCPDTBT-PC_70_BM is fixed to 80 nm throughout, which allows an appropriate balance between light absorption and carrier collection in the absorber according to previous studies^33^. Due to their promising performance in terms of electrical conductivity and transparency in visible light, the MoO_3_/Ag/MoO_3_ (DMD) multi-layer electrodes^34^ were considered as transparent conductive cathodes for the TOSCs. The 1D Ag gratings with a fixed thickness of 60 nm and the duty cycle (defined as f = W/P) of 0.5 were patterned on DMD electrodes. We performed full-field electromagnetic simulations with the finite-difference time domain (FDTD) method by using the commercial software of Lumerical FDTD solutions. Periodic boundary conditions were put on the x-direction, and perfectly matched layers on the upper and the lower boundaries. The refractive indices of ITO, PEDOT:PSS, PCPDTBT:PC_70_BM (1:3), and MoO_3_ are all extracted from previously published experimental results^32^,^35^.

Figure 2a shows the contour plots of the calculated transmission of multi-colored TOSCs as a function of the incident wavelength and the grating period. Spectral engineering in light transmission for the TOSCs is achieved by tuning the period of Ag gratings from 200 nm to 500 nm. The light transmission from the TOSCs is anticipated to be substantially increased when the forward-propagating wave constructively interferes with the resonantly coupled mode. For the multi-colored TOSCs, we are particularly interested in the wavelength region of 400–700 nm that containing most of the colors discernible to the human eye. As illustrated in Figure 2a, an obvious transmission band covers these wavelengths appearing in the map and the wavelength of its resonance position red-shifts with the increase of the grating period. The transmission spectra (normalized to the peak value of the resonance band) for six typical grating periods are shown in Figure 2b. In the shaded portions of the spectra, the color filtering properties of the TOSCs can be clearly identified as the light transmission is engineered by the dominate resonance bands that ranging from blue to red. Besides the resonant transmission bands, one can notice that there exist two abrupt changes in the transmission spectra which are mainly attributed to the Wood's anomalies (WA). The first one near the wavelengths given by λ ≈ n_air_P is transmission anomaly, and the other refers as reflection anomaly weakly interfering with the resonant bands at the wavelengths given by λ ≈ n_Glass_P (where n_air_ and n_Glass_ are the refractive indices of air and glass, respectively).

To clarify the origins of the light filtering characteristics of the proposed multi-colored TOSCs, the dispersion relations and modal profiles of the photonic modes supported by the multi-layered stacks in absence of any gratings (TOSCs) were numerically calculated. In the planar configurations of the TOSCs, the high-index layers of ITO, PCPDTBT-PC_70_BM and MoO_3_ have similar refraction index around 1.8 ~ 2 serving as a waveguide to couple incident wave into resonant modes. Figure 2c (left) shows the modal profile of the fundamental TM_0_ waveguide mode of the TOSC stacks. It is found that the thin PEDOT:PSS HTL and the inserted Ag layer in the MoO_3_ layer have only negligible influence on the waveguide modes. The intensity maximum of the modal profile is located in the active layer, which strongly resembles the magnetic field distribution along the stacking direction of the TOSC at the wavelength of the transmission peak from the FDTD simulations [Figure 2c (right)]. Additionally, as illustrated in Figure 2a, the white dotted line corresponds to the dispersion relations for the TM_0_ waveguide mode, which also matches well with the peak positions of the dominate transmission band. Therefore, the transmission peak observed in Figure 2a,b and its grating-assisted tunability can be attributed to the excitation of the TM_0_ waveguide modes through phase matching by grating couplers.

In the following, we will extend our considerations into the 2D numerical model of the multi-colored TOSCs, in which the array of Ag nanodiscs arranged into a hexagonal lattice was employed to achieve polarization-insensitive light filtering capacity as shown in figure 3a. The hexagonal nanodiscs have the same period along the three principal directions crossing at 60 degree, thus polarization-insensitive excitation of waveguide modes in the TOSCs can be anticipated owing to the three-fold symmetry of the GMR couplers. In our simulations, normal incidence with a plane wave polarized to one of the principal directions (x-polarization) of the hexagonal patterns was assumed. In order to achieve better color control in a broad range, meticulous optimization is performed by varying the thickness of the external MoO_3_ layer. In compare with the 1D grating structures, a slight thinner external MoO_3_ layer is required for the 2D structures to suppress the light transmission at short wavelengths to ensure high chromaticity for the red cells. Typical transmission spectra for the blue, green and red cells are plotted in the inset of figure 3b, in which well-defined narrow band light transmission can be observed. To provide a quantitative measure of the color perception of the multi-colored TOSCs, the chromaticity coordinates in accordance with the spectral transmittance were calculated and superimposed in the CIE 1931 xy color space diagram. This diagram enables us to quantitatively graph the saturation of a particular color. Figure 3b shows the predicted colors for the devices with periods ranging from 240 nm to 500 nm for three duty cycles of 0.3, 0.35 and 0.4. The chromaticity coordinate of the reference TOSC (without Ag nanodiscs) marked by the black pentagram is very close to the position of the white point (x = y = 0.33), which indicates a slight yellowish color for this semi-transparent cell because of their flat spectral transmission. In contrast, the incorporation of 2D Ag nanodiscs on the TOSCs will significantly alter the color perceptions of the cells. It is found that the proposed multi-colored TOSCs permit the color tuning span to encompass all primary colors by simply adjusting the period of the nanodiscs ranging from 240 nm to 500 nm. Meanwhile, we observed that the purities of the colors improved slightly with the increasing duty cycle of the nanodiscs, which is primarily attributed to the more suppressed off-resonance transmission for the cases with larger duty cycles.

Until now, we have mainly focused on the spectral engineering of the light transmission through the multi-colored TOSCs, in which the Ag nanostructures were considered as an efficient GMR coupler for tuning the resonance positions. It should be also noted that, the incorporation of metallic nanostructures on the rear side of the TOSCs may offer further advantages to enable strong light trapping in the photoactive layer^36^,^37^,^38^,^39^. To evaluate the light absorbability of the proposed multi-colored TOSCs and identify the possible light trapping mechanisms associated with the metallic nanostructures, we then present detailed comparisons between the multi-colored TOSCs (the period and duty cycle of the nanodiscs was set as P = 360 nm, f = 0.3, 0.35 and 0.4), opaque OSCs and semi-transparent OSCs (semi-TOSCs) in terms of their light transmission, reflection, parasitic absorption losses and light absorption in the photoactive layer. The calculated spectra are plotted in Figures 4a–4d. The reference cells with f = 0 and 1 represent the semi-TOSCs and opaque OSCs, respectively. In the absence of any metallic nanostructures, the semi-TOSCs with single-pass light absorption have high light transmission loss in the entire absorption region of the photoactive layer, leading to a poor light harvesting capacity. On the contrary, a continuous Ag layer with thickness of 60 nm fully blocks the light transmission from the opaque cells. Consequently, the light absorption in opaque OSC is almost doubled when compared to the semi-transparent cell as shown in Figure 4d. For the multi-colored TOSCs incorporated with periodic metallic nanostructures, Figure 4a clearly shows one distinct transmission peak around λ = 570 nm for each duty cycle, corresponding to the resonant guided mode. Compared with the semi-TOSCs, their optical absorption in the photoactive layer greatly increases due to the marked suppression of transmission loss at the off-resonance wavelengths, in which the Ag nanodiscs with minimal parasitic absorption function as an efficient back reflector (refer to Figure 4c, where the majority of the parasitic absorption is due to the loss in Ag structures).

It is very interesting to note that the multi-colored TOSCs preserve high absorbability comparable to the opaque OSCs (seen Figure 4d), although substantially more photons pass through the cells and contribute to their colored semi-transparency. From this perspective, the transmission loss is almost equally compensated by the enhanced light localization in photoactive layer, which suppresses reflection loss from the front surface of the multi-colored TOSC as shown in Figure 4b. At the resonance wavelength (around λ = 570 nm) of guided modes, we examine the magnetic field profiles within the multi-colored TOSCs for information about the mechanisms leading to enhanced light localization. As illustrated in Figure 4e, the magnetic field profiles show a typical feature of GMR that are apt to be confined in the high-index photoactive layers at the vicinity of two adjacent nanodiscs. With the strong onset of light localization, a reflection minimum occurs at the resonance wavelength, as shown in Figure 4b, indicating that the incident wave is partially absorbed by the waveguide layer and partially leaks out from the metallic nanostructures.

At the off-resonance wavelengths, the gain mechanism of light absorption in the multi-colored TOSCs is governed by the broadband scattering effect of the Ag nanodiscs. For illustration purposes, we then calculated the absorption cross-sections, forward and backward scattering cross-sections (normalized to the geometrical cross-section) of the Ag nanodiscs according to Mie theory^40^. Without losing generality, we focus on a simplified configuration comprising of an isolated Ag nanodisc on semi-infinite MoO_3_ substrate. As illustrated in Figure 4f, the Ag nanodisc with a diameter of 240 nm has minimal absorption cross-sections over a broad wavelength range, in agreement with the low parasitic absorption losses observed in Figure 4c. Additionally, it appears that the scattering in the backward direction is dominant for the entire wavelength range considered here. These findings suggest the fact that, the Ag nanodiscs with large backward scattering cross-sections offer advantages over the purely back reflector since the strong back scattered light acquires an angular spread in the photoactive layer that can effectively increase the optical path-length. As can be seen from the inset of Figure 4f, the electric-field distributions within the multi-colored TOSCs clearly show the evidence of strong backward scattering behavior of the Ag nanodiscs (P = 360 nm, f = 0.4). Since the scattering intensity profile is well localized within the photoactive layer, the light scattering effect arising from the Ag nanodiscs may considerably increase the performance of light trapping in multi-colored TOSCs.

Finally, a thorough evaluation of the performance of the multi-colored TOSCs is presented. For a solar cell, the short-circuit current density is an important parameter that determines the power-conversion efficiency. In order to quantify the absorption of the multi-colored TOSCs across the solar spectrum, we calculate the ultimate photocurrent under the assumptions of unity internal quantum efficiency with the expression^41^: 
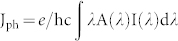
, where e is the elementary charge, h is Plank's constant, c is the speed of light, I(λ) is AM1.5G spectrum, and A(λ) is the absorptance of active layer. The J_ph_ calculated for the reference opaque OSCs and semi-TOSCs are 19.57 mA/cm^2^ and 12.36 mA/cm^2^, respectively. The normalized photocurrent for the multi-colored TOSCs (defined as the photocurrent ratio between multi-colored TOSCs and semi-TOSCs) is plotted in Figure 5a. In general, it is found that the normalized photocurrent increases steadily with the increasing of the duty cycle of metallic nanodics, and finally converges to the value of the opaque case (1.58). Due to the coexistences of light guiding and backward scattering effects, a maximum normalized photocurrent around 1.63 is observed for the multi-colored TOSC with P = 360 nm, f = 0.6, which surpass the opaque cell. Since there is a clear trade-off between light absorption performance and the level of the transparency in the semi-transparent solar cells, we then calculated the luminosity (the perceived brightness of a color) of our proposed multi-colored TOSCs. The luminosity for each cell shown in Figure 5b is defined as the integrated transmission spectrum over the whole visible spectrum, weighted by the photopic spectral response of a typical human eye. As expected, the multi-colored TOSCs containing nanodics with smaller duty cycle have higher luminosity, which is consistent with the dependence of light transmission on the duty cycles shown in Figure 4a. Meanwhile, it is observed that the luminosity of a multi-colored TOSC containing nanodics with a small or large P is lower than the cell with a moderate P around 350 nm. This can be understood by noting that human eye is more sensitive for green light.

To give a more direct sense of how the efficiencies and the color perceptions are related, the RGB colors which involve the information of brightness and chromaticity are calculated for the multi-colored TOSCs containing nanodics with the different periods and duty cycles, as illustrated in Figure 5c. Every square in this map represents a color pixel that displays distinct RGB color. Thus, totally 238 colors can be distinguished at such a “geometry resolution” (ΔP ~ 20 nm, ΔDuty cycle ~ 0.05). From the Figure 5a–c, the relationship between the colors and the cell efficiencies can be distinguished in a straightforward manner. TOSCs containing nanodiscs with small duty cycles have high luminosity but low in the efficiency and color purity, and the situation is reversed for the large duty cycles. For nanodiscs with moderate duty cycles 0.3 ~ 0.7, the calculated photocurrents of the multi-colored OSCs are around 42% ~ 63% higher than the semi-transparent OSCs, and different colors with clear perception ranging from blue to red can be accessed by tuning the period of the nanodiscs.

Using the similar design frameworks, our analysis can be extended to the inverted architectures using metallic nanostructures that embedded in the PEDOT:PSS layer, where similar color control can be expected under DMD-side illumination as illustrated in figure 6a. Due to the variation of dielectric environment of the metallic nanostructures, aluminum (Al) is found to perform better than the silver, allowing narrow-band light filtering. The configurations for the multi-layered stacks remain the same with the normal design (figure 3a), except for the thickness of PEDOT:PSS layer is increased to 80 nm. The thickness variation slightly modifies the dispersion characteristics of the multi-layered stacks, thereby the period range for assessing full-colors is increased (see figure 6a, RGB color map). A square array of Al nanocubes (50 nm in height) patterned on ITO layer are assumed to be fully encapsulated by PEDOT:PSS. In comparison with the normal structures, even higher photocurrent enhancement is obtained for the inverted multi-colored TOSCs as illustrated in figure 6b. The reason for this is that the reference inverted semi-TOSC (without Al nanocubes) has a relative low photocurrent around 8.27 mA/cm^2^. Meanwhile, from the figure 6c, it is found that the luminosities of the inverted design maintain at relative high levels for a duty cycle up to 0.8, which is much higher than the normal design with the same duty cycle. In this inverted architecture, the duty cycles ranging 0.4 ~ 0.8 is optimal choice for guaranteeing efficient color control, clear color perceptions and 80%–130% higher photocurrents than the semi-transparent OSCs. In view of the experimental realization of the above two structures, the scalable nanofabrication techniques like the nanoimprint^36^ and laser interference lithography^42^ are highly preferred. Considering the vulnerability of the polymers, the nanotransfer printing technique developed in our previous work^43^ that is capable of printing the metallic nanostructures directly onto the polymer-based substrate can be an optimum choice for the former.

In conclusion, our theoretical studies demonstrate that multi-colored semi-transparent organic solar cells (multi-colored TOSCs) employing metallic nanostructures as both light filtering and back scattering elements stand as a highly effective platform for achieving color control, clear color perceptions and high efficiency simultaneously. The two cell architectures illustrated in this paper show the wide parameter space available to the optimization the color and efficiency performance of the multi-colored TOSCs. This general design strategy can quite easily be extended to a variety of absorbing polymers with low band gap. Moreover, the multi-colored TOSCs have a prominent advantage over the multilayered thin-film designs: different colors can be spatially controlled on the same substrate by a single patterning process. It could serve as a dual-functional device with both integrated color filtering and photovoltaic power generation capabilities thus seem to be attractive for the self-powered colorful electronic displayer devices.

## Author Contributions

W.L. and C.Q. conceived the idea and designed the theoretical framework. W.L. wrote the main manuscript text and conducted the FDTD simulations. W.L. and J.L. calculated the modal characteristics of the multi-layer stacks. S.F. and S.S. did the Mie scattering calculations. Both, C.Q. and Y.Y. contributed for the editing of the manuscript. All authors discussed the results and reviewed the manuscript.

## Figures and Tables

**Figure 1:**
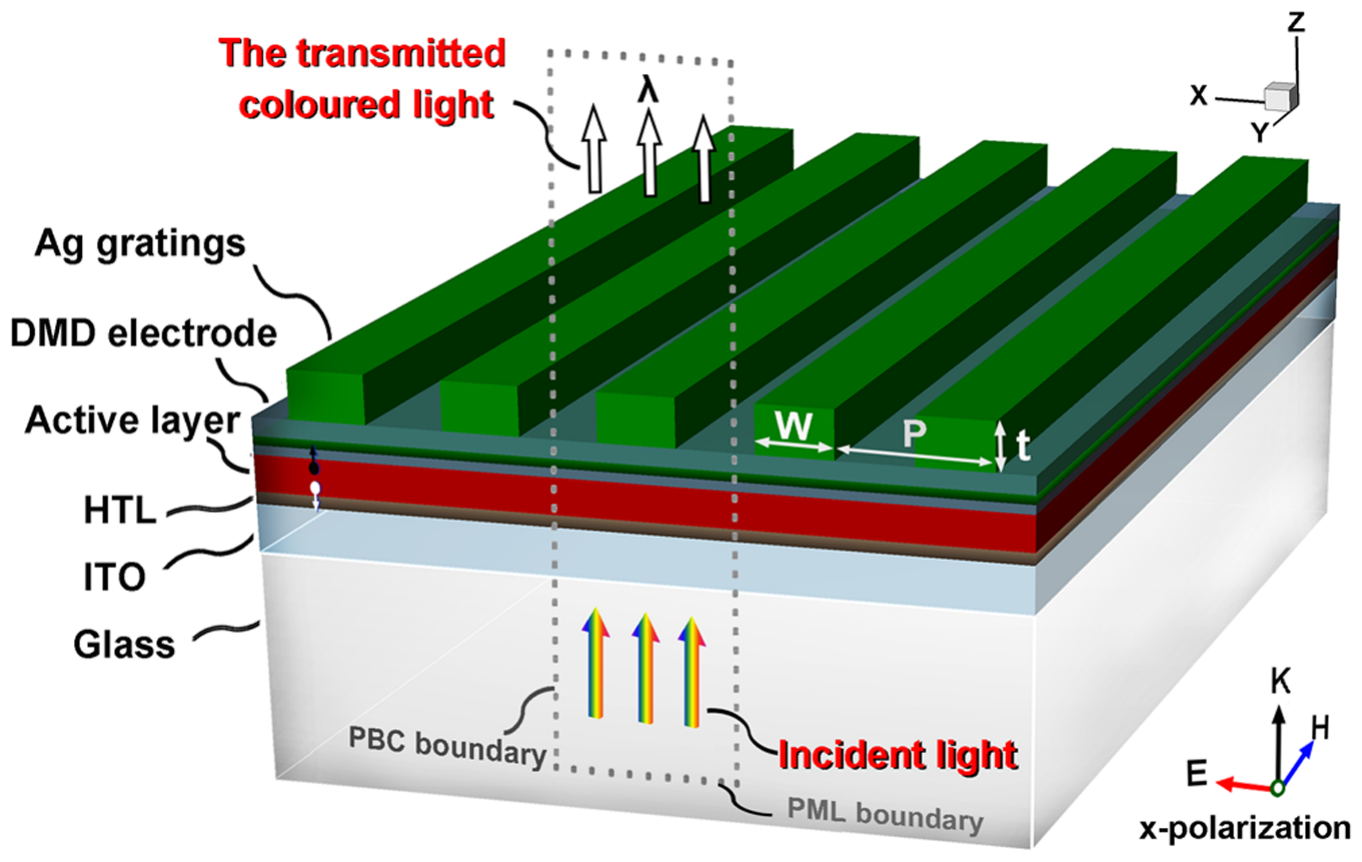
Schematic of multi-colored TOSCs comprised of a semi-infinite glass substrate, a 80 nm thick ITO anodes, 10 nm PEDOT:PSS hole transport layer (HTL), 80 nm PCPDTBT:PC_70_BM active layer, MoO_3_ (15 nm)/Ag (10 nm)/MoO_3_ (35 nm) DMD cathodes patterned with 1D Ag gratings (period P, width W and a fixed thickness t of 60 nm). The device is illuminated from the glass substrate at normal incidence with a plane wave polarized perpendicular to the grating grooves (TM-illumination).

**Figure 2:**
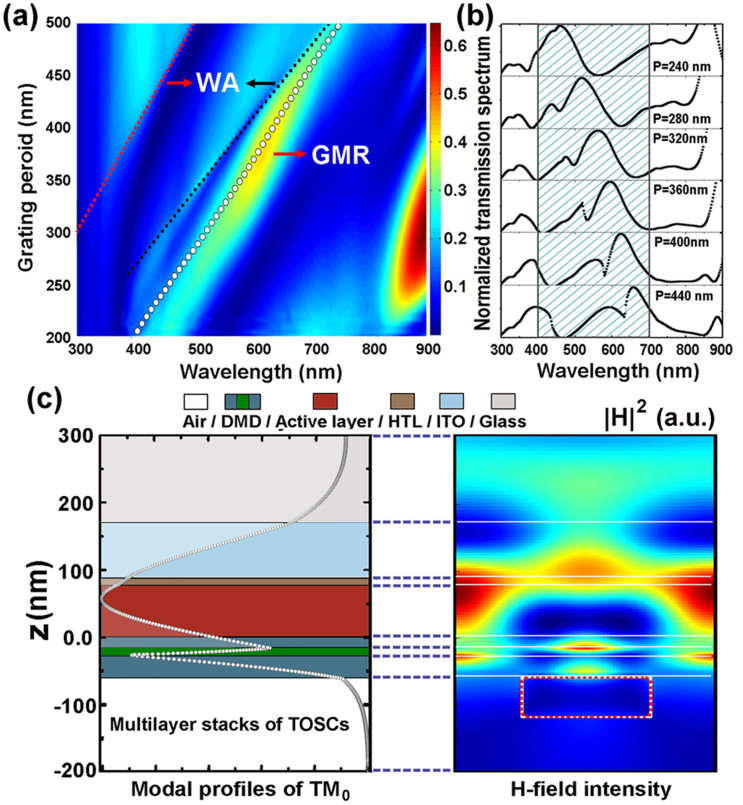
(a) Contour plots of transmission from the multi-colored TOSCs versus the incident wavelength and period of the Ag gratings for TM illumination, when the duty cycle of the gratings is set to be f = 0.5. The red and black dotted lines given by the relationships of λ ≈ n_air_P and λ ≈ n_Glass_P refer to transmission and reflection anomaly, respectively. The white dotted line corresponds to the dispersion relations (wavelength versus 2π/β, where β is propagation constant of the guided modes) for the fundamental TM_0_ GMR mode supported by the multilayer stacks of TOSCs. (b) Normalized transmission spectra of the multi-colored TOSCs with different grating periods. (c) Modal profiles of TM_0_ waveguide mode of the TOSCs multilayer stacks (left); Normalized magnetic field distributions inside the multi-colored TOSCs at the resonant wavelength (right).

**Figure 3:**
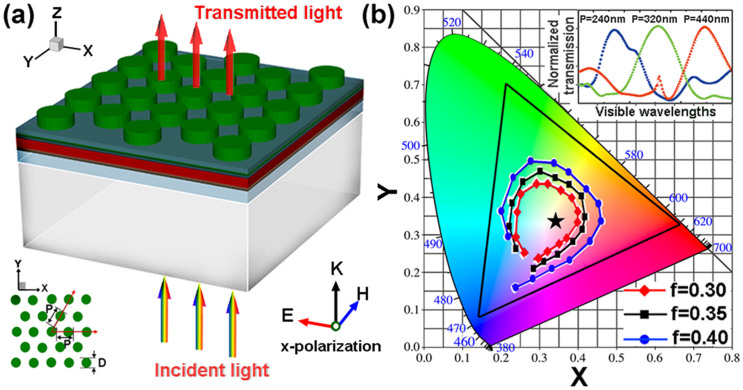
(a) Schematic of the 2D Ag nanodiscs patterned multi-colored TOSCs. The nanodiscs are characterized by their diameter D, period P and the duty cycle. The thickness configurations of the TOSCs remain the same as the 1D structure, except for the DMD electrode which becomes 15 nm/10 nm/25 nm. (b) CIE 1931xy Chromaticity coordinates represents for the predicted colors of the multi-colored TOSCs integrating the 2D Ag nanodiscs array with various periods (ranging from 240 nm to 500 nm in the clockwise direction of the curves) and duty cycle (defined as 
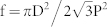
). The black pentagram represents the chromaticity coordinate of the light transmission from the reference TOSCs. The inset shows three typical transmission spectra (P = 240, 320 and 440 nm, f = 0.45) for the blue, green and red cells.

**Figure 4:**
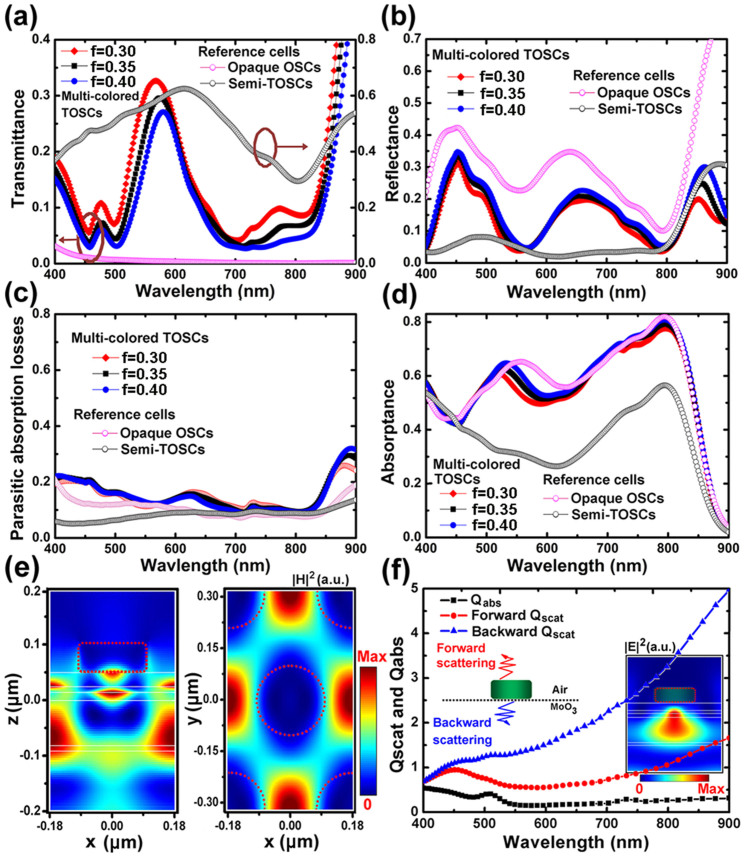
The calculated transmittance (a), reflectance (b), parasitic absorption losses in ITO, PEDOT:PSS, MoO3 and metal structures (c), and light absorption in the photoactive layer (d) for the multi-colored TOSCs (P = 360 nm, f = 0.3, 0.35 and 0.4) and the reference cells. Two reference cells with f = 0 and 1 represent the semi-transparent OSCs and opaque OSCs, respectively. (e) Normalized magnetic-field distributions inside the multi-colored TOSCs (f = 0.4) at the resonant wavelength (λ = 566 nm) in the x-z cutting plane located at the center of the nanodiscs (left), and in the x-y cutting plane located in the active layer (right). (f) The absorption cross-sections (Q_abs_), forward and backward scattering cross-sections (Q_scat_) of the Ag nanodiscs. The inset shows the normalized electric-field distributions of the multi-colored TOSCs (f = 0.4) at the wavelength of λ = 700 nm.

**Figure 5:**
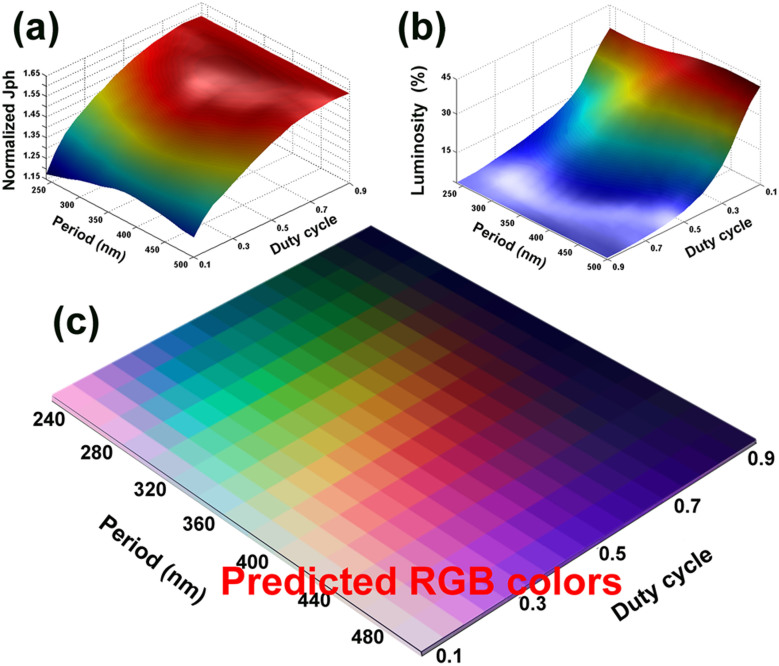
(a) The normalized photocurrent and (b) the luminosity of the multi-colored TOSCs as a function of P and duty cycle. (c) Predicted RGB colors for different periods and duty cycles.

**Figure 6:**
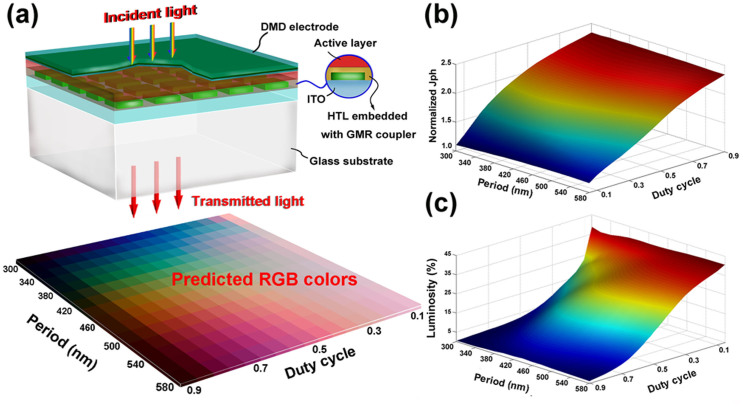
(a) Schematic of the inverted multi-colored TOSCs and Predicted RGB colors for different periods and duty cycles. The periodic Al nanocubes are characterized by their width W, period P and the duty cycle (W^2^/P^2^). (b) The normalized photocurrent and (c) the luminosity of the inverted multi-colored TOSCs as a function of P and duty cycle.
